# Primary Pulmonary Synovial Sarcoma in a 49-Year-Old Male

**DOI:** 10.7759/cureus.11899

**Published:** 2020-12-04

**Authors:** Sanket Shah, Praveen Sankrithi, Kunal Shah, Samir Dalia, Mohan Rudrappa

**Affiliations:** 1 College of Osteopathic Medicine, Kansas City University of Medicine and Biosciences, Kansas City, USA; 2 Internal Medicine/Hematology, Mercy Hospital, Joplin, USA; 3 Internal Medicine/Pulmonology, Critical Care Medicine, Mercy Hospital, Joplin, USA

**Keywords:** multimodal, pulmonary sarcoma

## Abstract

Sarcoma is a malignant tumor arising from the mesenchymal tissues such as striated skeletal and smooth muscles, adipose tissue, bone, cartilage, and synovial tissue. The synovial subset of primary pulmonary sarcoma is very rare and is only described in a handful of cases. Our case describes the diagnostic approach and treatment regimen for a 49-year-old male with no significant past medical history. The diagnosis of a primary pulmonary synovial sarcoma was made through the use of endobronchial ultrasound (EBUS), transbronchial needle aspiration (TBNA), histologic analysis, and immunostaining. The treatment utilized a multimodal approach including resection, chemotherapy, and radiotherapy.

## Introduction

Sarcoma is a malignant tumor arising from the mesenchymal tissues such as striated skeletal and smooth muscles, adipose tissue, bone, cartilage, and synovial tissue. Synovial sarcoma is a rare malignancy of mesenchymal origin constituting about 8% of all sarcomas [1]. Synovial sarcoma has the potential to arise anywhere in the body but almost 90% of all synovial sarcomas arise from the extremities with a high predilection for the knee joints. While the exact origin of synovial sarcoma is uncertain, it is believed to arise from the pluripotent mesenchymal cells located in various organs of the body. Additionally, primary pulmonary sarcomas can arise from the pluripotent mesenchymal cells in the pulmonary system. Primary pulmonary sarcomas are uncommon and account for less than 0.5% of all pulmonary malignancies [2]. The synovial subset of primary pulmonary sarcoma is very rare and is only described in a handful of cases.

## Case presentation

A 49-year-old male with no significant past medical problems presented in the emergency department (ED) with acute chest pain along with dyspnea that had been ongoing for three weeks. The chest pain was described as radiating to his upper left back, pleuritic and progressively worsening over the past two days. Family history was significant for mesothelioma in the paternal grandfather and pancreatic cancer in the paternal grandmother. Vital signs were stable except for mild tachycardia and complete blood count (CBC)/comprehensive metabolic panel (CMP) results were within normal limits.

**Figure 1:**
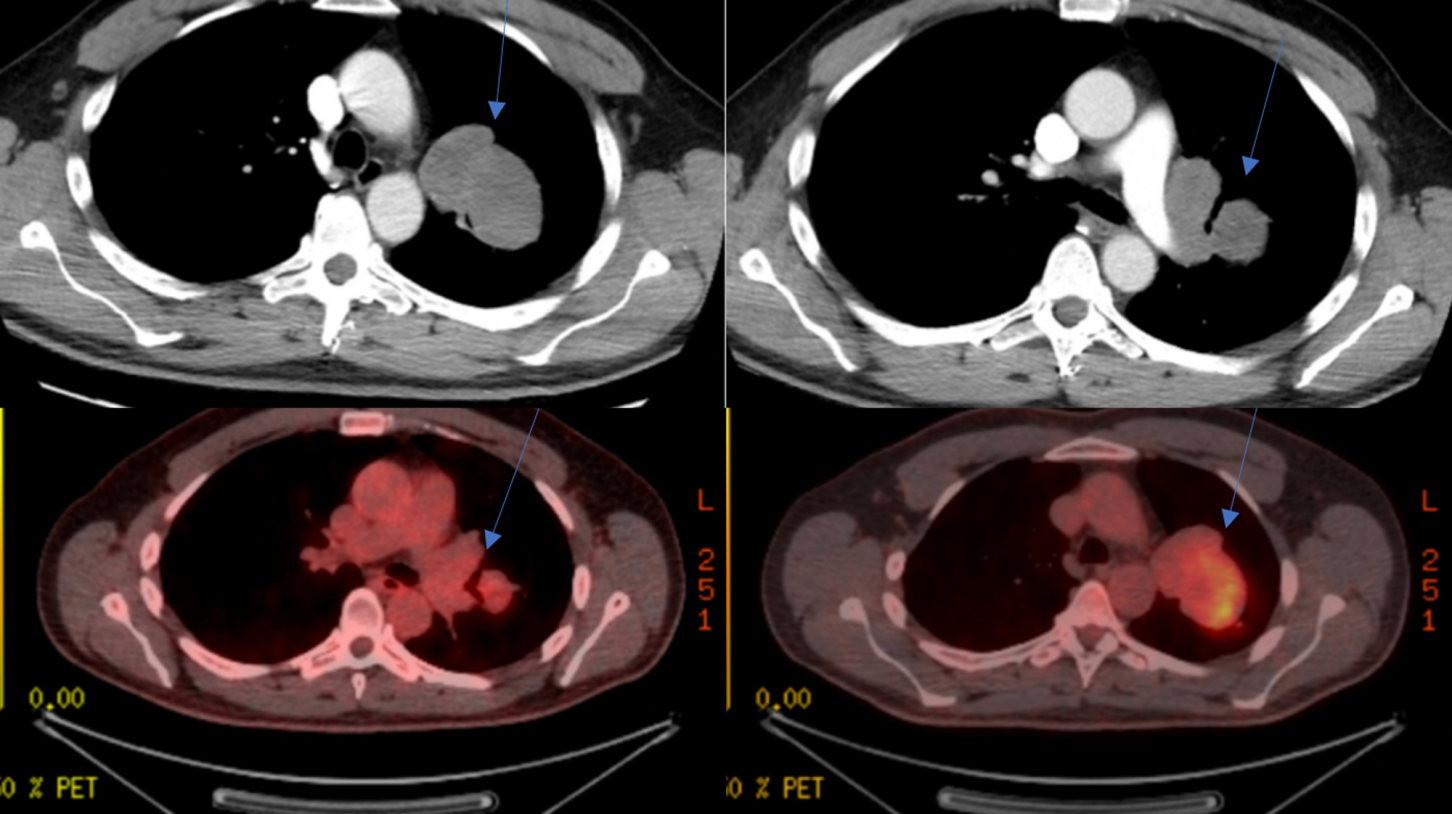
CT/PET scan results The images on the top left and top right show the bilobular, smooth borders of the sarcoma located in the left upper lobe of the lung. The images on the bottom left and bottom right detail the positron emission tomography (PET) scan results showing heterogeneous activity in the area of the mass. The blue arrows indicate the area of interest.

The chest X-ray in the ED showed an anterior mediastinal mass. Computed tomography (CT) revealed a large well-circumscribed bilobed low-density mass completely encasing but not obstructing or infiltrating the distal left main bronchus or the left upper lobe bronchial branches (Figure 1) and positron emission tomography (PET) showed heterogeneous activity in the area of the mass (Figure 1). No obvious hemorrhage or necrosis was noted. The mass measured 7.4 cm by 6.3 cm at its largest dimension. No obvious hilar or mediastinal lymphadenopathy was noted on the CT scan.

**Figure 2:**
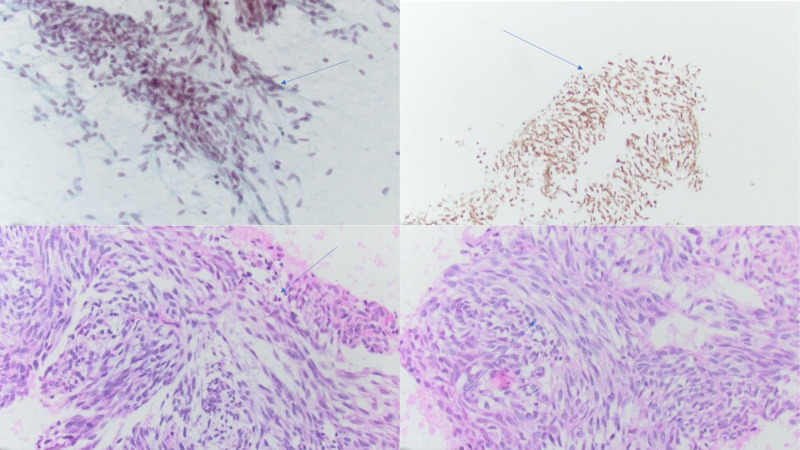
Histologic images obtained from the transbronchial needle aspiration (TBNA) sample The image on the top left demonstrates features of the monophonic synovial sarcoma. The image on the top right shows positive Bcl-2 immunostaining of spindle cells. The images on the bottom left/right show elongated spindle cells with oval nuclei and minimal cytoplasm. The arrows indicate areas of interest.

Due to the patient's history of smoking, primary lung cancer was considered and the patient underwent bronchoscopy with endobronchial ultrasound (EBUS). Bronchoscopy showed excellent cleanout of the left upper lobe bronchus with scant mucus but no obvious endobronchial mass. EBUS revealed a large hypoechoic mass in the left secondary carina. Transbronchial needle aspiration (TBNA) was done and the initial pathology report revealed a relatively non-specific spindle cell neoplasm. The biopsy samples were consistent with synovial cell sarcoma, and the histologic findings are detailed in Figure 2. Further imaging with PET scan and magnetic resonance imaging (MRI) of the brain revealed no metastatic lesions in the bones or the brain.

Immunostaining was positive for Bcl-2. Calretinin, CAM5.2, CD34, WT1 and AE1/AE3 profiles were negative. Samples sent out for diagnosis confirmation were positive for synovial sarcoma based on morphological findings and a positive t(X:18) )(p11;q11) translocation. Due to the marginal resectability of the tumor, the patient completed four cycles of chemotherapy with adriamycin and ifosfamide and had a good therapeutic response to the neoadjuvant therapy before surgery. A 5.7 cm x 4.8 cm x 6 cm tumor was removed along with two positive intrapulmonary lymph nodes. After surgery, the patient proceeded with radiation therapy. Unfortunately, the patient had a new subcutaneous abdominal nodule which was concerning for metastatic disease and is currently scheduled for a biopsy of the nodule for a possible metastatic lesion.

## Discussion

Sarcomas in the pulmonary system are most commonly secondary to metastasis from extrapulmonary sarcomas. Primary pulmonary sarcomas are rare. The mesenchymal tissue in the pulmonary system can differentiate into synovial sarcoma, leiomyosarcoma, or histiocytoma which are also the most commonly observed primary pulmonary sarcomas [3].

Primary pulmonary synovial sarcoma is a unique tumor as there is no synovial tissue located in the pulmonary system. The tumor usually arises from the pluripotent mesenchymal cells which then differentiate into synovial tissue. It is usually seen in the fourth or fifth decade of life with equal sex distribution. There is no predilection for the right or left side of the lung, but it is most commonly seen in the apex of the lungs [3]. Smoking is not a predisposing factor in the development of the tumor [4]. Primary pulmonary synovial sarcomas grow slowly and insidiously, which can often lead to delayed diagnosis and therapy. Clinical features and presentation of intrapulmonary tumors are mostly based on tumor location. The most common presenting symptoms are chest pain, cough, dyspnea, and occasionally hemoptysis [4]. The tumor tends to be large and, at times, can cause a partial or complete opacification of the lungs. Pleural effusion, cavitation, calcification, and lymphadenopathy are typically absent in patients with primary synovial sarcoma [5,6]. Most tumors are larger than 7 cm, causing compression of the surrounding tissue and making the site of origin difficult to discern. Bone involvement or mediastinal adenopathy is rarely reported [6]. 

A chest X-ray of a primary sternal sarcoma typically shows a well-defined uniform homogeneous mass lesion with round, lobulated borders. A CT scan of the chest with contrast typically shows heterogeneous enhancement due to the presence of a necrotic, cystic, or friable portion of the tumor interspersed with a solid enhancing component of the tumor [7]. On MRI, primary synovial sarcoma typically shows triple signal areas of bright, dark, and gray attenuation due to the presence of a tumor, hemorrhage, and necrosis, respectively [8].

Endobronchial ultrasound (EBUS) and transbronchial needle aspiration (TBNA) have been increasingly used to assist in diagnosis due to frequent tumor proximity to the major airways, as seen in our patient. On gross examination, the tumor appears gray-white or tan with a soft fleshy to firm rubbery consistency and is well-circumscribed, with or without a capsule. Necrosis and hemorrhage can also be observed within the tumor [9]. On histological examination, there are two main subtypes of synovial sarcoma, biphasic and monophasic. Poorly differentiated, monophasic epithelial, calcifying, and myxoid types of sinusoidal have also been described [9]. Monophasic synovial sarcoma, as observed in this patient, is the most common variant seen in primary synovial sarcoma. This is composed of relatively small and uniform spindle cells with ovoid, pale staining nuclei and inconspicuous nucleoli. The cytoplasm is scant and indistinct cell borders with intervening collagenous stroma give the appearance of nuclear overlapping [10]. Biphasic synovial sarcoma consists of both epithelial and spindle components. The epithelial cell component contains ovoid nuclei and has a cytoplasm that is organized in a glandular pattern [10].

Diagnosis is also aided by immunostaining. Monophasic synovial sarcomas usually stain positive for vimentin, Bcl-2, CD-99, TLE, FLi-1, and S-100. In our patient, immunostaining was positive for Bcl-2, and tests for calretinin, CAM5.2, CD34, WT1, and AE1/AE3 were negative. Cytogenetic testing for the t(X:18) )(p11;q11) translocation has high sensitivity and is considered to be the gold standard [11,12]. This translocation results in an SYT-SSX1 or SYT-SSX2 fusion transcript which was present in the patient confirming the diagnosis of synovial sarcoma [12].

There is no standard recommendation for the optimal management of patients with primary synovial sarcoma. When feasible, resection of the tumor as early as possible remains the standard of care followed by chemoradiation [13]. Synovial sarcoma is a relatively chemosensitive tumor that responds well to adriamycin alone or in combination with ifosfamide [14]. Randomized control trials and observational studies have shown that an ifosfamide based regimen improves the survival in extremity soft tissue sarcomas. A combination regimen of adriamycin and ifosfamide has been suggested for rapid symptom relief and in patients who are planning to have a curative resection. Radiation therapy alone or combined with surgery is used for achieving good local control of the disease [14].

Pazopanib, an oral vascular endothelial growth factor receptor inhibitor has been effectively used in patients with synovial sarcoma of any location. As most primary synovial sarcoma tumors also contain an SS18-SSx fusion oncogene, several new therapeutic agents targeting the oncogene have been developed and are currently in initial trials [14].

## Conclusions

Primary synovial sarcoma is an extremely rare intrathoracic tumor seen usually in young adults. When a primary tumor is in the chest, it usually presents with a large intrathoracic mass without any bone or mediastinal involvement. They can be accurately diagnosed with transthoracic EBUS and TBNA. Histologic analysis and immunostaining of tumor biopsy can help identify the tumor type. Most synovial sarcoma tumors also show a fusion oncogene which helps in obtaining an accurate diagnosis. Treatment is based on a multimodal approach including resection, chemotherapy, and radiotherapy.
